# What can we learn from noncoding regions of similarity between genomes?

**DOI:** 10.1186/1471-2105-5-131

**Published:** 2004-09-15

**Authors:** Thomas A Down, Tim JP Hubbard

**Affiliations:** 1 Wellcome Trust Sanger Institute, Wellcome Trust Genome Campus, Hinxton, Cambridge, CB10 1SA, UK

## Abstract

**Background:**

In addition to known protein-coding genes, large amounts of apparently non-coding sequence are conserved between the human and mouse genomes. It seems reasonable to assume that these conserved regions are more likely to contain functional elements than less-conserved portions of the genome.

**Methods:**

Here we used a motif-oriented machine learning method based on the Relevance Vector Machine algorithm to extract the strongest signal from a set of non-coding conserved sequences.

**Results:**

We successfully fitted models to reflect the non-coding sequences, and showed that the results were quite consistent for repeated training runs. Using the learned models to scan genomic sequence, we found that they often made predictions close to the start of annotated genes. We compared this method with other published promoter-prediction systems, and showed that the set of promoters which are detected by this method is substantially similar to that detected by existing methods.

**Conclusions:**

The results presented here indicate that the promoter signal is the strongest single motif-based signal in the non-coding functional fraction of the genome. They also lend support to the belief that there exists a substantial subset of promoter regions which share several common features including, but not restricted to, a relative abundance of CpG dinucleotides. This subset is detectable by a variety of distinct computational methods.

## Background

Since the publication of draft sequences for the human [1] and mouse [2] genomes, several groups have run large-scale comparisons of the sequences to detect regions of conserved sequence. An initial survey of these was published along with the draft mouse genome [2], with additional comparisons appearing since then [3]. Briefly, protein coding genes are – as we might expect – among the most strongly conserved regions, but homologous sequences can be found throughout the genome. In total, it is possible to align up to 40% of the mouse genome to human sequence [4], but it seems likely that at least some of this is just random "comparative noise" – regions of sequence which serve no particular purpose but which, purely by chance, have not yet accumulated enough mutations to make their evolutionary relationship unrecognisable. However, it is widely accepted that some of the noncoding-but-similar regions, especially those with the highest levels of sequence identity between the two species, are preferentially conserved because they perform some important function. It has been estimated that around 5% of the genome is under purifying selection [2], indicating that mutations in these regions have deleterious effects: a strong suggestion of some important function.

Here, we apply the Eponine Windowed Sequence (EWS) sequence analysis method method which uses a Relevance Vector Machine (RVM) [5] to extract a minimal set of short motifs which are able to discriminate between two sets of sequences: in this case, a positive set of conserved non-coding sequences and a negative set of randomly picked non-coding sequences. The EWS model is an adaption of the Eponine Anchored Sequence (EAS) model, first applied for transcription start site prediction in [6] and subsequently used to predict a range of additional biological features including translation start sites and transcription termination sites [A. Ramadass, unpublished] While EAS is designed to classify individual points in a sequence – a feature which allows the model to predict precise locations for features such as transcription start sites – EWS classifies complete blocks (windows) of sequence. The basis functions (inputs) of the RVM are sums of position-weight matrix scores [7] across the whole window.

## Results

We considered a set of alignments made by the blastz program [4] between release NCBI33 of the human genome and release NCBIM30 of the mouse genome. Since unprocessed blastz aligns around 40% of human sequence to the mouse genome, we chose to focus on the 'tight' alignments. These are a subset of alignments which are rescored and thresholded using a set of parameters given in [4], and cover only around 5.6% of the human genome – a proportion much closer to the fraction of bases thought to be under purifying selection [2].

**Figure 1:**
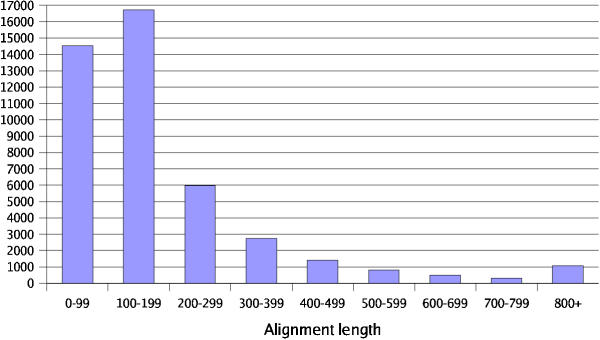
**Blastz alignments between human chromosome 6 and the mouse genome. **Histogram showing number of alignments covering human sequences of various lengths.

In total, the tight blastz set contained 787173 blocks of sequence with high-scoring alignments between the two genomes. We considered only those blocks assigned to human chromosome 6, a 170 Mb chromosome which has recently undergone manual annotation of gene structures and other features [8]. This chromosome included 44105 (5.6%) of the total alignments. These varied in length from 34 to 9382 bases, with a length distribution skewed towards relatively short alignments, as shown in figure 1.

Since we were interested in non-coding features of the genome, we ignored all regions where an alignment overlaps an annotated gene structure. This removed 20.8% of aligned bases. It is possible that some genes, and especially pseudogenes, have been missed by the annotation process, so we also removed portions covered by *ab initio *gene predictions from the Genscan program [9]. This eliminated an additional 4.3% of aligned bases. Finally, repetitive sequence elements annotated by the programs RepeatMasker [10] and trf [11] (5.9%) were removed from the working set. The remainder of the aligned regions were split into non-overlapping 200 base windows, ignoring any portions less than 200 bases. This gave a set of 13925 sequences which are well-conserved between human and mouse – and therefore likely to be functional – but which are very unlikely to be part of the protein-coding repertoire. These formed the positive training set for our machine learning strategy.

A negative training set of equal size was prepared by picking 200-base windows at random from the non-coding, non-repetitive portions of chromosome 6, using the same criteria to define repeats and coding sequence. While it is probable that this set also included some functional sequences, we would expect them to be represented at a substantially lower level than in the conserved set.

These two sets of sequence were presented to the Eponine Windowed Sequence machine learning system, as described in the methods section. Randomly chosen 5-base words were used as seed motifs, and three independent training runs were performed, each for 2000 cycles. The set of motifs used in model 1 is shown in table 1.

**Figure 2:**
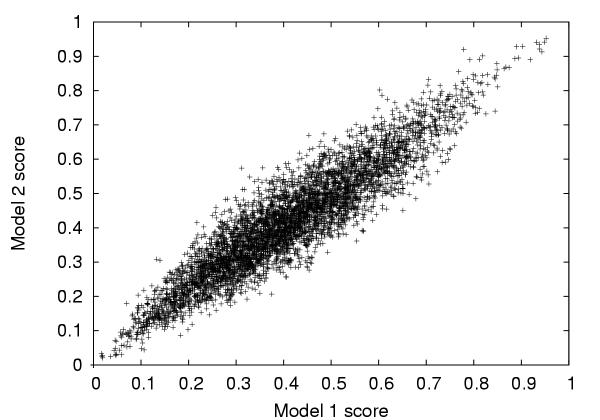
**Correlation of model scores. **Scatter plot showing the scores of EWS models 1 and 2 on a set of human sequences.

While the exact set of motifs used in the model varied somewhat from run to run, testing pairs of models on non-overlapping windows from a 1 Mb region of human chromosome 22 and plotting the scores showed that the model outputs were highly correlated (*e.g*. figure 2). We calculated the Pearson correlation coefficient for all pairs, and in all cases this was greater than 0.96. From this strong correlation, we concluded that any variations in the model were simply the result of the trainer picking one representative from a group of motifs which provide similar information.

**Figure 3:**
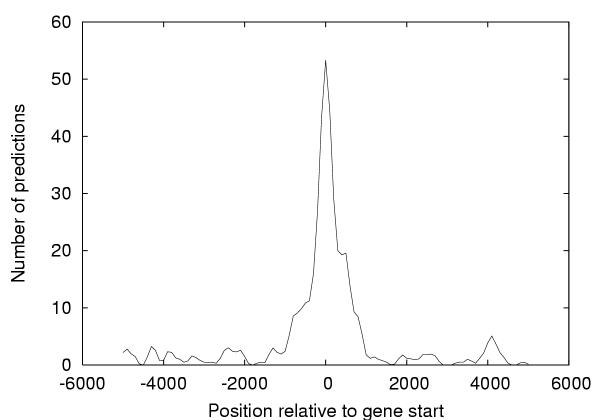
**Localisation of predictions. **Density of predictions from one of the homology models around known gene starts on human chromosome 22

We scanned genomic sequences using these models at a range of thresholds, and examined the results on the Ensembl genome browser [12] using a Distributed Annotation System [13] server. Visual inspection showed that many of the highest-scoring regions were localised near the start of genes. This prompted us to look at the distribution of high-scoring sequences with respect to the starts of a set of well-annotated genes. We considered the GD_mRNA genes from version 2.3 of the human chromosome 22 annotation. These are confidently annotated genes with experimental evidence as described in [14], which confirms at least the approximate location of the ends of the transcripts, and are independent from the chromosome 6 training data. Figure 3 shows the density of predictions with EWS scores ≥ 0.90 relative to the annotated 5' ends of these genes. This shows a strong peak of predictions close to the annotated starts, demonstrating that the model is predicting some sequences commonly located around the transcription start site of genes. Combining this observation with the fact that the model was trained from conserved (and therefore presumed functional) sequences, we believe that it is detecting signals found in the promoter regions of genes.

**Figure 4:**
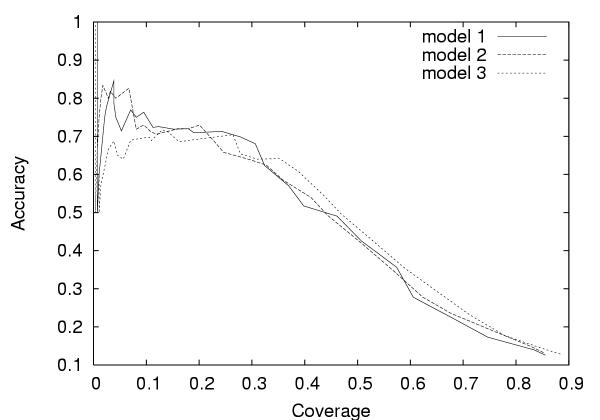
**Accuracy and coverage of TSS prediction. **Plots of Accuracy vs. coverage at a range of score thresholds (ROC curves) for three independently trained homology models

Evaluation of promoter-prediction methods on a large scale is a difficult exercise, since there are no large pieces of genomic sequence for which we can be certain we know the complete set of transcribed regions, and even in the case of well-known genes we often do not know the precise location at which transcription begins. In [6], we developed a pseudochromosome, derived from release 2.3 of the chromosome 22 annotation. As described above, this includes a subset of 284 experimentally verified gene structures. The pseudochromosome was constructed to include these genes while omitting all other annotated genes (which could be substantially truncated). We considered predictions (groups of one or more overlapping windows which all have scores greater than some chosen threshold) to be correct if they lie withing 2 kb of an annotated gene start, and false otherwise. Plotting accuracy (proportions of predictions which are correct) against coverage (proportion of transcript starts which are detected by one of the correct predictions) gives a Receiver Operating Characteristic (ROC) curve. Using this criterion, a totally random set of predictions would be given an accuracy of around 0.07. ROC curves are plotted for the three independently trained models in figure 4. Firstly, this shows that predictive performance for all three models is rather similar. It also shows that they can function as accurate promoter predictors, with accuracy rising to a plateau of around 0.7, much higher than expected for random predictions.

**Figure 5:**
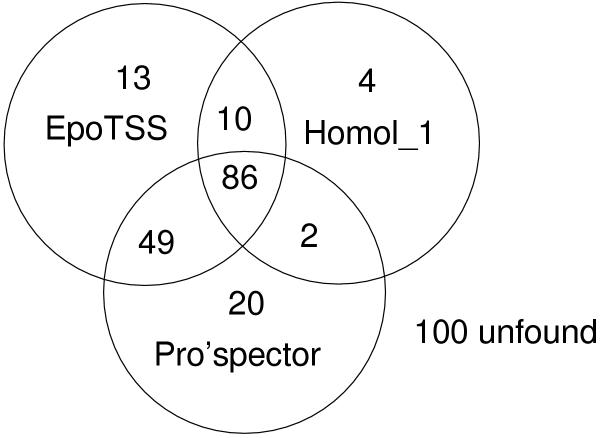
**Comparison of TSS prediction methods. **Sets of pseudochromosome promoters correctly predicted by three different prediction methods: EponineTSS [6] with a score threshold of 0.999, PromoterInspector (labelled "Pro'spector"), and the homology-EWS model 1 with a score threshold of 0.91 ("Homol_1").

We picked model 1 for further study. Using a score threshold of 0.91, this gives an accuracy of 0.68 and a coverage of 0.31. We compared the set of genes correctly detected by this model to two other methods: firstly, the EponineTSS predictor described in [6], and secondly, the published results from the PromoterInspector program [15]. PromoterInspector results were mapped to pseudochromosome coordinates using the procedure described in [6]. Figure 5 shows how the set of promoters detected by these three distinct methods overlaps. There are clearly strong correlations between all three methods. In particular, at this threshold the EWS homology model detects 98 promoters which were found by at least one of the other methods, but only 4 novel promoters.

**Figure 6:**
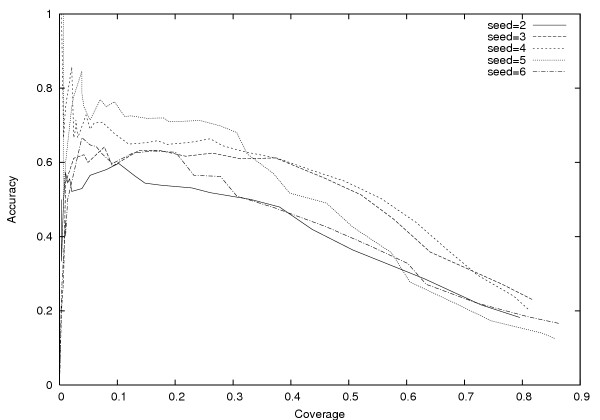
**Effect of seed-word size of learning. **Accuracy vs. coverage plots for models trained using seed word lengths of 2 to 6 bases.

We investigated the robustness of the signal learned by this process by retraining models with a variety of seed word sizes, from 2 to 6 bases. During training, motifs can be trimmed to lengths shorter than that of the seed words (down to a minimum of 2 bases) but can never grow longer than the seed word size. When evaluated on the pseudochromosome, the resulting models always showed a preference for regions around gene starts, regardless of word length, as shown in figure 6. However, the accuracy was reduced when using short seed words – particularly words of length of 2. The best accuracy was seen for a seed word length of 5, and decreased somewhat for words of length 6.

**Figure 7:**
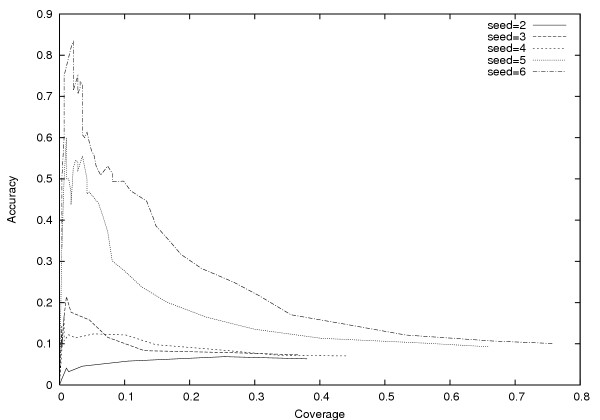
**Effect of excluding CpG dinucleotides. **Accuracy vs. coverage plots for models trained using a range of seed-word sizes, with all CpG dinucleotides removed from both training and test data.

This suggests that a large fraction (but not all) of the information learned by these models can be encoded in dinucleotide frequencies. It is well known that many transcription start sites are close to regions of relatively high CpG dinucleotide composition (CpG islands) [16]. To investigate the contribution that CpG dinucleotides make to our models, we deleted all CpG dinucleotides from the training data, then re-evaluated the resulting models on the pseudochromsome (also with CpG dinucleotides removed), as shown in figure 7. Perhaps not surprisingly, dinucleotide models now show very little tendency to detect gene starts. However, as the word size increases, the preference for gene starts gradually increases, until a seed size of 6 gives an accuracy comparable to that see when CpG dinucleotides are included, although the maximum coverage before accuracy begins to drop rapidly is somewhat lower. Broadly similar results are seen if CpG dinucleotides are randomly replaced with other dinucleotides.

## Conclusions

We have shown here that, when presented with a set of non-coding sequences which are strongly conserved between human and mouse, a simple motif-oriented machine learning system consistently builds models which are able to detect a substantial fraction of human promoter regions with good accuracy. This strongly suggests that this promoter signal represents the most widely used motif-based signal in functional non-coding sequence. While the model learned here can clearly be applied for the purpose of genome-wide promoter annotation, in practise existing methods offer better coverage and (in the case of the EponineTSS predictor) predictions for the precise location of the transcription start site.

It is interesting that the promoter model learned by this technique detected substantially the same set of promoters as found by the EponineTSS and PromoterInspector methods. It has previously been remarked that these two methods detect similar sets [6], but this could perhaps be explained by the fact that both methods were initially derived from similar sets of known promoter sequences (in both cases, training data was extracted from the EPD database [17]. In the case of the homology models described here, there is no connection with EPD, or any similar set of known promoters: the training data was picked purely on the basis of its high similarity to corresponding portions of the mouse genome. These results therefore support the alternate view that there is a particular 'easily detected' subclass of promoter sequences.

One distinct group of promoters, which previous results show may correspond to this easily detected family, is the set of promoters associated with CpG islands [16]. However, while a number of the motifs listed in table 1 are G/C rich and/or contain the CpG dinucleotide, by no means all of the motifs match this description, and indeed one motif containing CpG has a negative weight in the linear model – its presence in a sequence will reduce the model's output score – while some A/T rich motifs have positive weights. We therefore believe that the signals detected here are significantly more complex than a simple over-representation of CpG dinucleotides. Experiments with smaller seed-word sizes support this assumption: while dinucleotide-based models were also able to predict promoter regions, the accuracy was lower than for models including longer motifs. Finally, we show that while the predictive capacity of dinucleotide models is largely eliminated once CpG dinucleotides are removed from the sequence, models including longer words are still able to make correct promoter predictions in many cases. So while CpG dinucleotides are an important contribution to the promoter signal, they are clearly not the only component.

## Methods

### Genomic sequence and annotation

Human genome sequence release NCBI33 and mouse genome release NCBIM30 were extracted from Ensembl databases [12], which also contained gene predictions from Genscan [9] and repeat data from RepeatMasker [10] and trf [11]. Curated annotation of gene structures on human chromosome 6 was obtained from the Vega database [18]. Vega and Ensembl data was extracted directly from the SQL databases using the BioJava toolkit with biojava-ensembl extensions [19].

### Genome alignments

Human-mouse genome alignments were generated by the blastz alignment program. These were subsequently re-scored and filtered to give a 'tight' set of high-confidence alignments, as described in [4]. We downloaded the tight alignment set from the UCSC genome website [20].

### Pseudochromosome for testing promoter-finding methods

A 16.3 Mb pseudochromosome sequence was produced based on version 2.3 of the curated annotation for human chromosome 22. This includes all the experimentally-validated gene structures and their upstream regions, while omitting regions containing genes that are predicted but not fully verified. In the case of a pair of divergent genes where one has been verified and the second has not, their shared upstream region was cut at the midpoint. More information about pseudochromosome construction is given in [6].

### Eponine Windowed Sequence learning

The Eponine Windowed Sequence (EWS) model is designed by analogy to the Eponine Anchored Sequence model first described in [6], but rather than targeting individual points in the sequence, it is designed to classify small regions or windows of a sequence, based purely on their own sequence content.

The EWS model uses the Relevance Vector Machine [5] algorithm to drive the training process. Relevance Vector Machines solve classification and regression problems by building Generalised Linear Models (GLMs) as weighted sums of a "working set" of basis functions. During the training process, those basis functions which are not informative are given weights close to zero and eventually discarded from the working set. To explore very large sets of possible basis functions, it is possible to add extra basis functions during the course of the training process [6].

The "sensors" of the EWS model are DNA position-weight matrices [7], which make convenient models of short sequence motifs. When using weight matrices to analyse sequence windows, we sum the weight matrix probability scores for all possible positions within the sequence. Normalising for the length of the sequence being inspected and the size of the PWM, the basis functions of the model take the form:


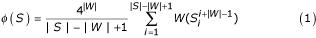


where *W*(*s*) is the probability that sequence *s *was emitted by weight matrix *W*, |*S*| is the sequence length, |*W*| is the weight matrix length, and 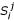 denotes a subsequence from *i *to *j*.

An initial set of basis functions is proposed by taking all possible DNA motifs of a specified length (typically 5) and generating weight matrices which preferentially recognise these motifs. As the relevance vector machine trainer removes non-informative basis functions from the working set, they are replaced by applying one of the following sampling strategies to a basis function picked randomly from the working set:

• Generate a new weight matrix in which each column is a sample from a Dirichlet distribution with its mode equal to the weights in the corresponding column of the parent weight matrix.

• Generate a new weight matrix one column shorter than the parent by removing either the first of the last column.

By using these sampling rules, the trainer is able to explore motif space. The process of generating candidate motifs using these rules then selecting the most informative using the RVM can be seen as a form of genetic algorithm.

## Authors' contributions

TD and TH conceived and designed this study, and analysed results. TD implemented the Eponine machine learning system and drafted the manuscript. All authors read and approved the final manuscript.

## Figures and Tables

**Table 1:** Motifs used in EWS homology model 1. The entries in this table show consensus sequences of the weight matrices used in the model (note that it is possible for two distinct weight matrices to have the same consensus sequence). Motifs are listed in both forwards and reverse-complement orientation, and the two sections of the table indicate whether that motif is given a positive or negative weight in the learned linear model.

Postive		Negative	
Forward	Reverse	Forward	Reverse
gtca	tgac	tacgt	acgta
tattg	caata	gggca	tgccc
tgcca	tggca	gtca	tgac
ggca	tgcc	acaat	attgt
tacgt	acgta	gggc	gcccc
gtact	agtac	tact	agta
taac	gtta	cctcc	ggagg
ttt	aaa	ggca	tgcc
acaat	attgt	tattg	caata
caatt	aattg	tattg	caata
cagc	gctg	aaatt	aattt
cag	ctg	caat	attg
cggat	atccg	gtat	atac
aaatt	aattt	ccagg	cctgg
gctcg	cgagc	catg	catg
ggc	gcc	act	agt
		taagg	cctta
		aaaaa	ttttt

## References

[B1] The Genome International Sequencing Consortium (2001). Initial sequencing and analysis of the human genome. Nature.

[B2] The Mouse Genome Sequencing Consortium (2002). Initial sequencing and comparative analysis of the mouse genome. Nature.

[B3] Xuan Z, Wang J, Zhang M (2002). Computational comparison of two mouse draft genomes and the human golden path. Genome Biology.

[B4] Schwartz S, Kent W, Smit A, Zhang Z, Baertsch R, Hardison R, Haussler D, Miller W (2003). Human-Mouse Alignments with BLASTZ. Genome Res.

[B5] Tipping M (2000). Sparse Bayesian learning and the relevance vector machine. Journal of Machine Learning Research.

[B6] Down T, Hubbard T (2002). Computational Detection and Location of Transcription Start Sites in Mammalian Genomic DNA. Genome Res.

[B7] Bucher P (1990). Weight matrix descriptions of four eukaryotic RNA polymerase II promoter elements derived from 502 unrelated promoter sequences. Journal of Molecular Biology.

[B8] Mungall A, Palmer S, Sims S, Edwards C, Ashurst J, Wilming L, Jones M, Horton R, Hunt S, Scott C, Gilbert J, Clamp M, Bethel G, Milne S, Ainscough R, Almeida J, Ambrose K, Andrews T, Ashwell R, Babbage A, Bagguley C, Bailey J, Banerjee R, Barker D, Barlow K, Bates K, Beare D, Beasley H, Beasley O, Bird C, Blakey S, Bray-Allen S, Brook J, Brown A, Brown J, Burford D, Burrill W, Burton J, Carder C, Carter N, Chapman J, Clark S, Clark G, Glee C, Clegg S, Cobley V, Collier R, Collins J, Colman L, Corby N, Coville G, Culley K, Dhami P, Davies J, Dunn M, Earthrowl M, Ellington A, Evans K, Faulkner L, Francis M, Frankish A, Frankland J, French L, Garner P, Garnett J, Ghori M, Gilby L, Gillson C, Glithero R, Grafham D, Grant M, Gribble S, Griffiths C, Griffiths M, Hall R, Halls K, Hammond S, Harley J, Hart E, Heath P, Heathcott R, Holmes S, Howden P, Howe K, Howell G, Huckle E, Humphray S, Humphries M, Hunt A, Johnson C, Joy A, Kay M, Keenan S, Kimberley A, King A, Laird G, Langford C, Lawlor S, Leongamornlert D, Leversha M, Lloyd C, Lloyd D, Loveland J, Lovell J, Martin S, Mashreghi-Mohammadi M, Maslen G, Matthews L, McCann O, McLaren S, McLay K, McMurray A, Moore M, Mullikin J, Niblett D, Nickerson T, Novik K, Oliver K, Overton-Larty E, Parker A, Patel R, Pearce A, Peck A, Phillimore B, Phillips S, Plumb R, Porter K, Ramsey Y, Ranby S, Rice C, Ross M, Searle S, Sehra H, Sheridan E, Skuce C, Smith S, Smith M, Spraggon L, Squares S, Steward C, Sycamore N, Tamlyn-Hall G, Tester J, Theaker A, Thomas D, Thorpe A, Tracey A, Tromans A, Tubby B, Wall M, Wallis J, West A, White S, Whitehead S, Whittaker H, Wild A, Willey D, Wilmer T, JM W, Wray P, Wyatt J, Young L, Younger R, Bentley D, Coulson A, Durbin R, Hubbard T, Sulston J, Dunham I, J R, Beck S (2003). The DNA sequence and analysis of human chromosome 6. Nature.

[B9] Burge C, Karlin S (1997). Prediction of complete gene structures in human genomic DNA. Journal of Molecular Biology.

[B10] Smit A, Green P RepeatMasker. http://ftp.genome.washington.edu/RM/RepeatMasker.html.

[B11] Benson G (1999). Tandem repeats finder: a program to analyze DNA sequences. Nucleic Acids Res.

[B12] Hubbard T, Barker D, Birney E, Cameron G, Chen Y, L C, Cox T, Cuff J, Curwen V, Down T, Durbin R, Eyras E, Gilbert J, Hammond M, Huminiecki L, Kasprzyk A, Lehvaslaiho H, Lijnzaad P, Melsopp C, Mongin E, Pettett R, M P, Potter S, Rust A, Schmidt E, Searle S, Slater G, Smith J, Spooner W, Stabenau A, Stalker J, Stupka E, Ureta-Vidal A, I V, Clamp M (2002). The Ensembl genome database project. Nucleic Acids Res.

[B13] Distributed Annotation System. http://www.biodas.org/.

[B14] Collins J, Goward M, Cole C, Smink L, Huckle E, Knowles S, Bye J, Beare D, Dunham I (2003). Reevaluating Human Gene Annotation: A Second-Generation Analysis of Chromosome 22. Genome Res.

[B15] Scherf M, Klingenhoff A, Freeh K, Quandt K, Schneider R, Grote K, Frisch M, Gailus-Durner V, Seidel A, Brack-Werner R, Werner T (2001). First pass annotation of promoters on human chromosome 22. Genome Res.

[B16] Cross S, Bird A (2002). The Ensembl genome database project.. Nucleic Acids Res.

[B17] Perier R, Praz V, Junier T, Bonnard C, Bucher P (2000). The Eukaryotic Promoter Database (EPD). Nucleic Acids Res.

[B18] Vega Genome Browser. http://vega.sanger.ac.uk/.

[B19] BioJava. http://www.biojava.org/.

[B20] UCSC Genome Bioinformatics. http://genome.cse.ucsc.edu/.

